# Association of rs2062323 in the *TREM1* gene with Alzheimer's disease and cerebrospinal fluid‐soluble TREM2

**DOI:** 10.1111/cns.14129

**Published:** 2023-02-23

**Authors:** Zuo‐Teng Wang, Yan Fu, Shi‐Dong Chen, Yu‐Yuan Huang, Ya‐Hui Ma, Yan‐Jiang Wang, Lan Tan, Jin‐Tai Yu

**Affiliations:** ^1^ Department of Neurology, Qingdao Municipal Hospital Qingdao University Qingdao China; ^2^ Department of Neurology, Qingdao Municipal Hospital, College of Medicine and Pharmaceutics Ocean University of China Qingdao China; ^3^ Department of Neurology and Institute of Neurology, Huashan Hospital, State Key Laboratory of Medical Neurobiology and MOE Frontiers Center for Brain Science, Shanghai Medical College Fudan University Shanghai China; ^4^ Department of Neurology and Centre for Clinical Neuroscience, Daping Hospital Third Military Medical University Chongqing China

**Keywords:** ADNI, Alzheimer's disease, polymorphism, sTREM2, *TREM1*

## Abstract

**Introduction and aims:**

Genetic variations play a significant role in determining an individual's AD susceptibility. Research on the connection between AD and *TREM1* gene polymorphisms (SNPs) remained lacking. We sought to examine the associations between *TREM1* SNPs and AD.

**Methods:**

Based on the 1000 Genomes Project data, linkage disequilibrium (LD) analyses were utilized to screen for candidate SNPs in the *TREM1* gene. AD cases (1081) and healthy control subjects (870) were collected and genotyped, and the associations between candidate SNPs and AD risk were analyzed. We explored the associations between target SNP and AD biomarkers. Moreover, 842 individuals from ADNI were selected to verify these results. Linear mixed models were used to estimate associations between the target SNP and longitudinal cognitive changes.

**Results:**

The rs2062323 was identified to be associated with AD risk in the Han population, and rs2062323T carriers had a lower AD risk (co‐dominant model: OR, 0.67, 95% CI, 0.51–0.88, *p* = 0.0037; additive model: OR, 0.82, 95% CI, 0.72–0.94, *p* = 0.0032). Cerebrospinal fluid (CSF) sTREM2 levels were significantly increased in middle‐aged rs2062323T carriers (additive model: β = 0.18, *p* = 0.0348). We also found significantly elevated levels of CSF sTREM2 in the ADNI. The rate of cognitive decline slowed down in rs2062323T carriers.

**Conclusions:**

This study is the first to identify significant associations between *TREM1* rs2062323 and AD risk. The rs2062323T may be involved in AD by regulating the expression of TREM1, TREML1, TREM2, and sTREM2. The TREM family is expected to be a potential therapeutic target for AD.

## Graphical abstract

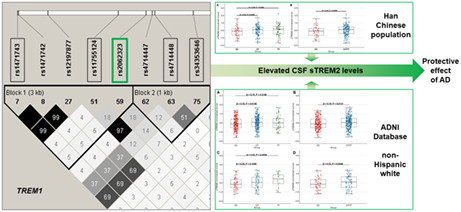

## 1 INTRODUCTION

Previous studies indicated that the worldwide prevalence of dementia will increase ninefold by 2050.^1^ Alzheimer's disease (AD) is a chronic disease with a long preclinical period (about 20 years), and it is the most common type of dementia.^2^, ^3^ About 95% of all AD patients worldwide belong to the late‐onset AD (LOAD) subtype. However, its pathogenesis is unclear, and the development of effective diagnostic and therapeutic tools has been hindered. Genetic studies have confirmed that LOAD is a polygenic and heterogeneous disease. And 60–80% of its risk depends on genetic factors.^1^ Therefore, screening and verifying LOAD susceptibility or pathogenic genes using genetic techniques is one of the research priorities in this field.

The triggering receptor expressed on the myeloid cells 1 (*TREM1*) gene is located in human chromosome 6p21.1, and immediately adjacent to the *TREM2* gene.^4^, ^5^ Monocytes/macrophages and blood neutrophils are the principal effector cells of human TREM1 in innate responses.^5^ TREM1 and TREM2 activate myeloid cells by signaling through the adaptor protein DAP12.^5^ The TREMs family is not only involved in the regulation of microglia phagocytosis and amyloid clearance, but also plays an indispensable role in the neuroinflammatory response of AD.^6^, ^7^, ^8^ Replogle and colleagues reported one common variant of the *TREM1* gene (rs6910730G) associated with AD pathology and aging‐related cognitive decline.^9^ One study found a significant decrease in TREM1 expression levels on the surface of peripheral blood monocytes from rs6910730G carriers.^10^ Phagocytosis of Aβ pathology was also significantly diminished by peripheral blood monocytes from rs6910730G carriers.^10^ In addition, the TREM1 can facilitate the microglial phagocytosis of Aβ, is associated with immune responses in AD.^10^, ^11^ These findings suggested that TREM1 can serve as a potential therapeutic target for AD.^12^ However, one study investigated the association between rs6910730G and AD (17,008 AD cases and 37,154 controls) using the International Genomics of Alzheimer's Project (IGAP) dataset and did not find any significant association between rs6910730G and AD susceptibility.^13^ Therefore, the association between *TREM1* gene polymorphism and AD risk needs further investigation. Moreover, it is necessary to further explore the underlying mechanisms between *TREM1* and AD susceptibility using human biological samples.

## 2 METHODS

### 2.1 Study population

A total of 1951 participants who were Han Chinese were gathered from Qingdao Municipal Hospital, Chongqing Daping Hospital, and Huashan Hospital. All eligible participants were required to be of Han Chinese origin and aged from 40 to 90 years old. The AD diagnosis was based on the criteria of the National Institute of Neurological and Communicative Diseases and Stroke and the Alzheimer's Disease and Related Disorders Association (NINCDS–ADRDA).^14^ The criteria for exclusion were: (1) family history of genetic diseases, (2) major neurological disorders, epilepsy, infection of the central nervous system, head trauma, or other neurodegenerative diseases (e.g., Parkinson's disease), (3) major psychological disorders, (4) severe systemic diseases (e.g., malignant tumors). Neuropsychological evaluations were performed on each subject using a structured questionnaire and an electronic medical record system. All physicians involved in the assessment have undergone standardized training. Blood samples from all participants were collected for testing. Cerebrospinal fluid (CSF) samples from 503 participants were collected for AD biomarker testing. Written informed consent was obtained from all participants or authorized representatives. The study's design was approved by the Institutional Review Board of all participating institutions (KY2020‐1161, Medical Research Ethics‐2021‐No.51, and 2017‐Temporary Audit‐No.26‐Fast).

In addition, 842 individuals (non‐Hispanic white) from Alzheimer's Disease Neuroimaging Initiative (ADNI) database (adni.loni.usc.edu) were selected to verify the associations between the target SNP and biomarkers. ADNI's goal is to investigate clinical, imaging, genetic, and biochemical AD biomarkers. Adult participants range in age from 55 to 90. The website http://www.adniinfo.org has comprehensive information.^15^, ^16^, ^17^

### 2.2 Construction of linkage disequilibrium (LD) blocks and selection of tag‐SNPs

The 1000 Genomes Project is a transnational cooperation project that contains whole‐genome sequencing information for 26 populations from the Americas, Europe, South Asia, East Asia, and Africa. We selected 1000 Genomes Project Han Chinese in Bejing Data (reference: Human Genome grch37) and used haploview 4.1 (Hardy–Weinberg equilibrium *p*‐value > 0.05, MAF > 0.1, LD threshold *r* ^2^ > 0.8) to analyze the SNPs locus on *TREM1* gene (6:41267385‐41286692).^18^ Finally, 10 tag‐SNPs inside *TREM1* were chosen based on the collected SNP information and LD blocks created by Haploview software (Table S2 and Figure S1). In addition, we also performed LD analysis using the Southern Chinese Han Chinese data (Figure S2) and European data (Figure S3) to verify the selected SNPs.

### 2.3 Genotyping

Fasting peripheral venous blood (4–5 mL) was collected using anticoagulant tubes. The QIAamp DNA Blood Mini Kit (Qiagen, Hilden, Germany) was utilized to extract DNA from blood samples in the Han Chinese population. Participants' DNA was subjected to genotyping using Infinium Asian Screening Array. ADNI samples were genotyped with the Human 610‐Quad BeadChip, Illumina Human Omni Express BeadChip, and Illumina Omni 2.5 M BeadChip. One SNP in *TREM1*, rs2062323, was analyzed for this study. *APOE* and genome‐wide genotyping data were obtained from this database (adni.loni.usc.edu). The *APOE* genotypes were determined using rs429358 and rs7412.

### 2.4 Measurement of AD core biomarkers

CSF was collected from participants in a standardized manner via lumbar puncture.^19^ Within 2 h, the CSF was transported to the lab and centrifuged at 2000*g* for 10 min. Prior to testing, the thaw/freeze cycle was limited to 2 cycles. Enzyme‐linked immunosorbent assay (ELISA) was used to quantify CSF Aβ42 (INNOTEST®; Fujirebio, Ghent, Belgium), T‐tau (INNOTEST®; Fujirebio, Ghent, Belgium), p‐tau (INNOTEST®; Fujirebio, Ghent, Belgium), and soluble TREM2 (sTREM2) (Abcam, no. Ab224881). All ELISA measurements are performed by experienced technicians who strictly follow the manufacturer's instructions. Clinical information is not disclosed to testers. Duplicate measurements were taken on samples and standards, and the average of the duplicate measurements was used for statistical analysis. All antibodies and plates are from a single batch to exclude variability between batches. In addition, the intra‐batch coefficient of variation (CV) was <5%, and the inter‐batch CV was <15%.

Alzheimer's Disease Neuroimaging Initiative provides sTREM2 data based on two platforms. One of the sTREM2 data is based on the MSD platform and has been comprehensively described in previous publications.^20^, ^21^, ^22^ The corrected values were used and are available in the ADNI database as variables “MSD_sTREM2corrected”. Furthermore, partial sTREM2 was tested at Washington University in St Louis. (developed in‐house: WU platform, Piccio group). The corrected values were used and are available in the ADNI database as variables “WU_sTREM2corrected.” The raw values are provided as pg/mL. The intraplate CV for CSF sTREM2 was <10%.

### 2.5 Statistical analysis

The baseline demographic characteristics (age, gender, APOE4 carriage status, and MMSE score) were compared using ANOVA and chi‐square tests. Logistic regression was utilized to analyze the relationship between target SNP and AD risk. Three genetic models, including co‐dominant, dominant, and additive models, were applied. All risk analysis models were corrected for age, sex, and APOE4 carriage status. Finally, Bonferroni correction was employed in the setting of multiple comparisons: *p*‐value < 0.005 was considered significant.

All continuous variables were normalized using the Box‐Cox transformations (R software “car” package) and standardized using the z‐scale transformations (R software “scale” package). We used multiple linear regression models to analyze the association between the target SNP and AD CSF biomarkers. The analysis used three genetic models (co‐dominant, dominant, and additive). All models were corrected for age, sex, and APOE4 status. In addition, we investigated the interaction of age, sex, and APOE4 carriage status on the association of rs2062323 with AD CSF biomarkers. Based on the results of the interaction, we further performed a subgroup analysis.

We included only non‐Hispanic white individuals in the ADNI replication cohort. The CSF sTREM2 (MSD and WU platform) data were depolarized (mean ± 3‐fold standard deviation), and the data were normalized and standardized using the R software “car” and “scale” packages. We used multiple linear regression models to analyze the association between the target SNP and CSF sTREM2. Three genetic models (co‐dominant, dominant, and additive) were utilized in the analysis. All models were corrected for age, sex, and APOE4 status. In addition, we investigated the interaction of age, sex, and APOE4 carrying status on the relationship between rs2062323 and sTREM2. Finally, we used linear mixed models to explore the effects of target SNP on longitudinal changes in cognition.

The above data were analyzed using SPSS software (version 22.0) and R software (version 3.6.1). We used the International Genomics of Alzheimer's Project (IGAP) to query the information related to our target SNP. In addition, expression quantitative trait loci (eQTL) analyses were conducted using multiple publicly available datasets (Genotype‐Tissue Expression (GTEx), https://gtexportal.org/home/snp/rs2062323) in human brain tissues and the whole blood.^23^

## 3 RESULTS

### 3.1 Characteristics of the study population

We included 870 cognitively normal individuals and 1081 AD patients to study the association between candidate SNPs and AD risk. Table 1 shows the demographic characteristics of these Han Chinese subjects. We studied the relationship between target SNP loci and AD biomarkers in 503 cognitively normal Han Chinese populations. The demographic characteristics of these 503 individuals are presented in Table 3. In addition, we included 676 non‐demented and 166 AD participants in the ADNI replication cohort. The demographic characteristics are shown in Tables S5 and S7.

**TABLE 1:** Baseline demographic characteristics of participants included.

Characteristic	HC (*n* = 870)	AD (*n* = 1081)	*p*
Age (years, mean ± SD)	64.41 ± 10.96	65.49 ± 9.95	0.025
Sex (female, %)	455 (52.3)	626 (57.9)	0.013
*APOE ε4* (yes, %)	152 (17.5)	454 (42.0)	<0.001
MMSE score (mean ± SD)	27.22 ± 2.96	15.43 ± 7.30	<0.001

*Note*: ANOVA, non‐parametric Kruskal‐Wallis H test, and Chi‐squared test were used to compare the baseline demographic and clinical characteristics.

Abbreviations: AD, Alzheimer's disease; MMSE, Mini‐Mental State Exam; SD, standard deviation.

### 3.2 Allele frequencies and genotype distributions of the tag‐SNPs

The genotype distribution of the target SNPs is shown in Tables S1 and S2. We identified a separate genetic locus rs2062323 on the *TREM1* that was significantly associated with AD risk in the Han Chinese population.

### 3.3 Association between tag‐SNPs within the TREM1 gene and the risk of AD

People carrying the rs2062323T allele had a significantly lower risk of AD (Table 2). *p*‐values < 0.005 after Bonferroni correction was considered significant. No statistically significant results were found at other SNP loci. There was no statistical significance for other SNPs.

**TABLE 2:** Associations between tag‐SNPs and the risk of sporadic Alzheimer's disease.

Tag‐SNPs	Genetic modelsa^a^	OR value	95% CI	*p*‐Value^b^
rs1471743	Codominant	0.94	0.75–1.19	0.6136
		1.68	0.80–3.70	0.1769
	Dominant	0.98	0.79–1.23	0.8838
	Additive	1.03	0.84–1.26	0.7975
rs11755124	Codominant	1.18	0.96–1.43	0.1107
		1.42	1.05–1.93	0.0243
	Dominant	1.22	1.01–1.48	0.0368
	Additive	1.19	1.03–1.36	0.0157
rs2062323	Codominant	0.78	0.62–0.97	0.0257
		0.67	0.51–0.88	**0.0037**
	Dominant	0.74	0.60–0.92	0.0058
	Additive	0.82	0.72–0.94	**0.0032**
rs4714448	Codominant	0.96	0.76–1.20	0.6986
		2.33	1.13–5.17	0.0279
	Dominant	1.02	0.82–1.28	0.8287
	Additive	1.09	0.89–1.33	0.3948
rs34353646	Codominant	0.98	0.78–1.24	0.8630
		2.34	1.13–5.20	0.0269
	Dominant	1.05	0.84–1.32	0.6656
	Additive	1.11	0.91–1.36	0.2960
rs6914090	Codominant	1.06	0.88–1.29	0.5382
		1.41	0.98–2.05	0.0693
	Dominant	1.11	0.92–1.34	0.2693
	Additive	1.13	0.97–1.31	0.1109
rs2234242	Codominant	1.21	0.99–1.47	0.0550
		0.77	0.53–1.11	0.1613
	Dominant	1.13	0.94–1.36	0.2048
	Additive	1.02	0.88–1.18	0.8106
rs6909482	Codominant	0.97	0.78–1.21	0.7967
		0.80	0.61–1.04	0.0987
	Dominant	0.92	0.75–1.13	0.4064
	Additive	0.90	0.79–1.03	0.1181
rs6910301	Codominant	1.22	1.00–1.48	0.0486
		0.84	0.59–1.19	0.3269
	Dominant	1.14	0.95–1.38	0.1556
	Additive	1.04	0.90–1.20	0.6293
rs6939973	Codominant	0.93	0.74–1.18	0.5529
		2.34	1.09–5.42	0.0353
	Dominant	1.00	0.80–1.25	0.9845
	Additive	1.06	0.87–1.30	0.5475

*Note*: All models were adjusted by age, sex, and APOE ε4.

Abbreviations: CI, confidence interval; OR, odds ratio.

^a^ Assuming M represents major allele and m represents minor allele, each genetic model can be described as follows: codominant: M/m vs M/M and m/m vs M/M, two OR values were listed from top to bottom in corresponding columns; dominant: (m/m + M/m) vs M/M; additive: m/m and M/m were weighed 2 and 1 respectively to M/M.

^b^ The given *p* values were not corrected by Bonferroni correction. Figures in bold indicate the retained association after Bonferroni correction.

**TABLE 3:** Baseline demographic characteristics of participants included.

Characteristic	HC (*n* = 503)
Age (years, mean ± SD)	62.84 ± 9.39
Sex (female, %)	266 (52.9)
Education (years, mean ± SD)	9.36 ± 4.45
*APOE ε4* (yes, %)	73 (14.5)
MMSE score (mean ± SD)	27.73 ± 2.38
CSF sTREM2 (pg/mL)	18688.99 ± 6924.02
CSF Aβ42 (pg/mL)	267.04 ± 142.37
CSF tau (pg/mL)	197.96 ± 94.52
CSF ptau (pg/mL)	43.17 ± 13.63

Abbreviations: AD, Alzheimer's disease; ADAS, Alzheimer's Disease Assessment Scale; ADNI_EF, Alzheimer Disease Neuroimaging Initiative‐executive function; ADNI_MEM, Alzheimer Disease Neuroimaging Initiative‐memory; CSF, cerebrospinal fluid; MMSE, Mini‐Mental State Exam; NFL, Neurofilament Light; NPI, Neuropsychiatric Inventory; SD, standard deviation.

**TABLE 4:** Multiple regression results for associations of CSF sTREM2 with rs2062323 stratified by age.

Genetic models^a^	CSF sTREM2 (Mid‐life)			CSF sTREM2 (Late‐life)		
	*n*	Mean ± SD	β, *p*‐value	*n*	Mean ± SD	β, *p*‐value
Codominant						
rs2062323^CC^	61	17009.38 ± 6511.57	reference	35	19961.54 ± 6749.44	reference
rs2062323^CT^	134	18367.54 ± 6571.81	**0.30, 0.0390**	85	20051.28 ± 7439.57	−0.03, 0.900
rs2062323^TT^	61	18765.97 ± 7184.54	**0.36, 0.0340**	45	18255.69 ± 6979.32	−0.31, 0.1740
Dominant						
rs2062323^CC^	61	17009.38 ± 6511.57	reference	35	19961.54 ± 6749.44	reference
rs2062323^CT/TT^	195	18492.18 ± 6753.31	**0.32, 0.0209**	130	19429.73 ± 7306.85	−0.13, 0.5110
Additive						
rs2062323^CC^ (0)	61	17009.38 ± 6511.565	**0.18, 0.0348**	35	19961.54 ± 6749.444	−0.16, 0.1510
rs2062323^CT^ (1)	134	18367.54 ± 6571.808		85	20051.28 ± 7439.574	
rs2062323^TT^ (2)	61	18765.97 ± 7184.54		45	18255.69 ± 6979.324	

*Note*: All models were adjusted by age, sex, APOE ε4, education (years), and MMSE score. Later‐life (>65 y) versus Mid‐life (≤65 y).

Abbreviations: Aβ, amyloid‐beta; AD, Alzheimer's disease; AV45, 18F‐AV45 amyloid‐PET; CSF, cerebrospinal fluid; SD, standard deviation; SNP, Single nucleotide polymorphism; sTREM2, soluble TREM2.

^a^ Assuming M represents major allele and m represents minor allele, each genetic model can be described as follows: codominant: M/m vs M/M and m/m versus M/M, two OR values were listed from top to bottom in corresponding columns; dominant: (m/m + M/m) vs M/M; additive: m/m and M/m were weighed 2 and 1, respectively, to M/M.

*p* values that are statistically significant are shown in bold.

**TABLE 5:** Multiple regressions results for associations of CSF sTREM2 with rs2062323 in ADNI.

Genetic modelsa^a^	CSF sTREM2 (non‐demented)			CSF sTREM2 (AD)		
	*n*	Mean ± SD	β, *p*‐value	*n*	Mean ± SD	β, *p*‐value
Codominant						
rs2062323^CC^	337	3867.45 ± 1783.10	reference	70	3932.31 ± 1876.94	reference
rs2062323^CT^	288	4190.77 ± 1963.62	**0.19, 0.0136**	53	4466.13 ± 2096.72	0.26, 0.1040
rs2062323^TT^	51	4152.04 ± 2084.20	0.15, 0.3150	12	5234.24 ± 2503.34	0.53, 0.0539
Dominant						
rs2062323^CC^	337	3867.45 ± 1783.10	reference	70	3932.31 ± 1876.94	reference
rs2062323^CT/TT^	339	4184.95 ± 1979.09	**0.19, 0.0131**	65	4607.39 ± 2181.96	**0.33, 0.0448**
Additive						
rs2062323^CC^ (0)	337	3867.45 ± 1783.10	**0.13, 0.0323**	70	3932.31 ± 1876.94	**0.26, 0.0252**
rs2062323^CT^ (1)	288	4190.77 ± 1963.62		53	4466.13 ± 2096.72	
rs2062323^TT^ (2)	51	4152.04 ± 2084.20		12	5234.24 ± 2503.34	

*Note*: All models were adjusted by age, sex, APOE ε4, education (years) and MMSE score. Later‐life (>65 years) versus Mid‐life (≤65 years).

Abbreviations: Aβ, amyloid‐beta; AD, Alzheimer's disease; AV45, 18F‐AV45 amyloid‐PET; CSF, cerebrospinal fluid; sTREM2, soluble TREM2; SD, standard deviation; SNP, Single nucleotide polymorphism.

^a^ Assuming M represents major allele and m represents minor allele, each genetic model can be described as follows: codominant: M/m vs M/M and m/m vs M/M, two OR values were listed from top to bottom in corresponding columns; dominant: (m/m + M/m) vs M/M; additive: m/m and M/m were weighed 2 and 1, respectively, to M/M.

*p* values that are statistically significant are shown in bold.

### 3.4 Association between tag‐SNP and CSF AD biomarkers

**FIGURE 1:**
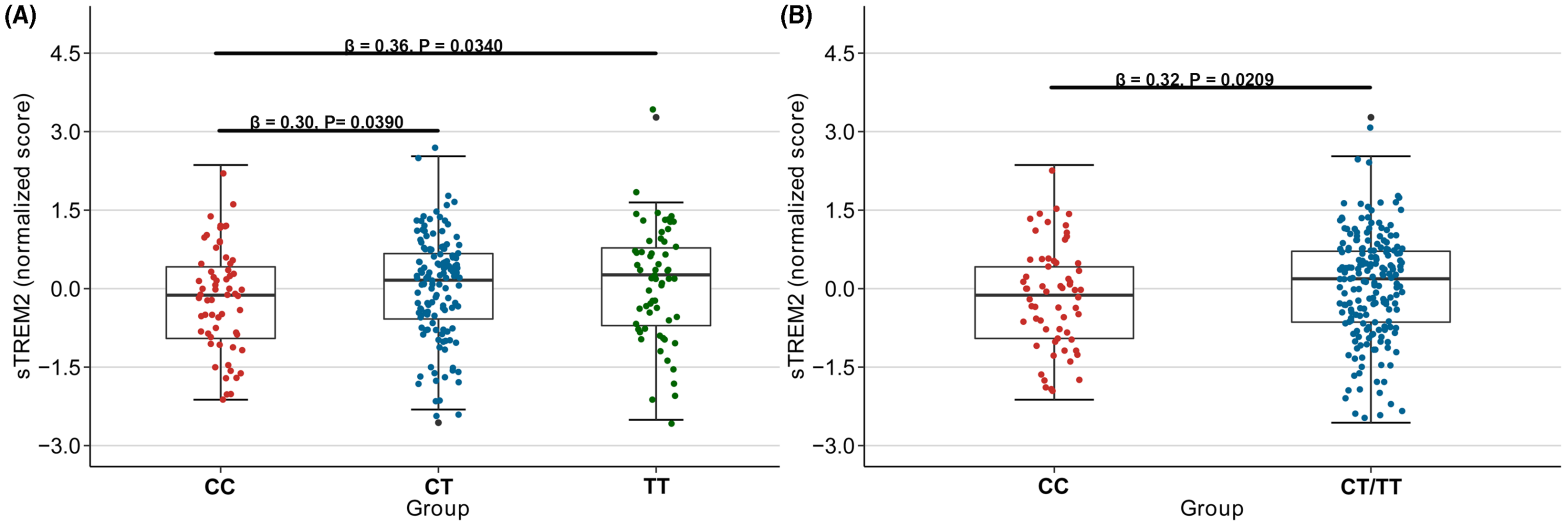
Associations of rs2062323 genotypes with CSF sTREM2 in middle‐aged (≤65 years) Han Chinese population. We categorized the rs2062323 into four subgroups: CC, CT, TT, and CT/TT. We found that CSF sTREM2 was significantly higher in rs2062323T carriers.

In the total population, no significant associations between the rs2062323 and AD CSF biomarkers were found in either the co‐dominant, dominant, or additive models (Table S3). In addition, interaction analysis revealed an interaction between the rs2062323 and CSF sTREM2 with age (β = −0.0308, *p* = 0.0436) (Table S4). CSF sTREM2 levels were significantly higher in middle‐aged (≤65 years) Han Chinese carrying rs2062323T compared to non‐carriers in age‐based subgroup analyses (Figure 1 and Table 4). We found no significant correlations in the elderly population (>65 years) (Table 4).

### 3.5 Replication in the ADNI cohort

**FIGURE 2:**
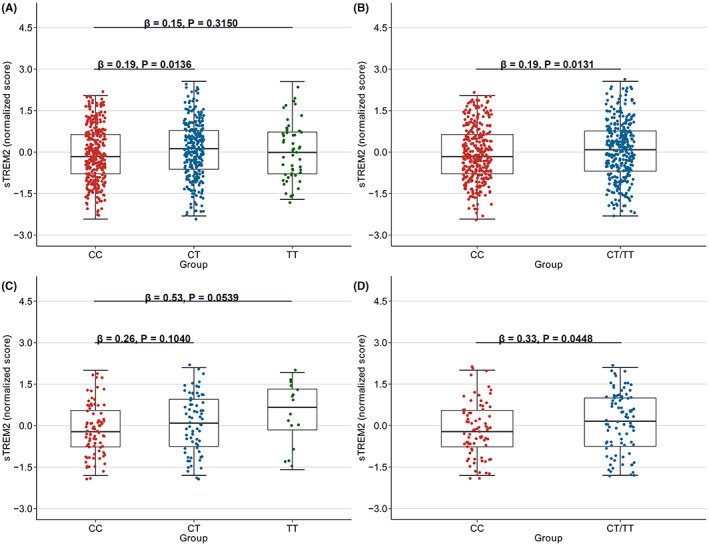
Associations of rs2062323 genotypes with CSF sTREM2 in ADNI. We categorized the rs2062323 into four subgroups: CC, CT, TT, and CT/TT. We found that CSF sTREM2 (MSD platform) were significantly higher in CT and CT/TT groups in the non‐demented population (A and B). In addition, CSF sTREM2 was significantly higher in CT/TT groups in AD patients (C and D).

We found significantly higher CSF sTREM2 (MSD and WU platform) levels in people carrying rs2062323T in the ADNI database. For MSD CSF sTREM2, significant associations existed in the non‐demented population (Figure 2A, Figure 2B, and Table 5) and AD patients (Figure 2C, Figure 2D, and Table 5). For WU CSF sTREM2, significant associations existed both in the non‐demented population and AD patients (Table S8).

**FIGURE 3:**
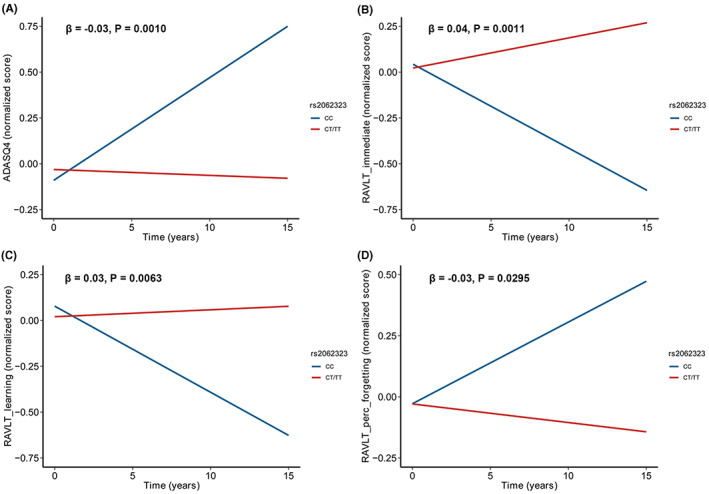
Associations of rs2062323 genotypes with longitudinal cognitive changes in non‐demented population in ADNI. The rate of cognitive decline was significantly slower in rs2062323T carriers compared to non‐carriers. ADAS_Q4: β = −0.03, *p* = 0.0010 (A); RAVLT_immediate: β = 0.04, *p* = 0.0011 (B); RAVLT_learning: β = 0.03, *p* = 0.0063 (C); RAVLT_Percent_Forgetting: β = −0.03, *p* = 0.0295 (D).

In addition, we performed interaction analyses in both diagnostic groups. Age, sex, and APOE4 genotype had no significant effect on the relationship between the rs2062323 and CSF sTREM2 (Tables S6 and S9). We also investigated longitudinal cognitive changes in a non‐demented population. The results showed that the rate of cognitive decline was significantly slower in rs2062323T carriers compared to non‐carriers (Figure 3).

### 3.6 Functional annotation of rs2062323

The IGAP did a meta‐analysis using the existing Genome‐Wide Association Studies (GWAS) dataset in 2013. The stage 1 meta‐analysis included 17,008 AD cases and 37,154 controls of European ancestry. Using these summary results, we identified that the rs2062323 was significantly associated with AD risk (effect allele, T, β = −0.0381, *p* = 0.03086).^24^ Moreover, multi‐tissue eQTL analyses showed that rs2062323 could significantly influence *TREM1*, *TREM2*, and *TREML1* expression in the whole blood and specific brain regions (including basal ganglia, hypothalamus, and hippocampus) (Figures S4–S6). *TREM1*, *TREM2*, and *TREML1* expression levels are elevated in rs2062323T carriers (Figures S4–S6).

## 4 DISCUSSION

In conclusion, we identified a genetic variant in *TREM1* is associated with the risk of AD in the Han Chinese population. The rs2062323T carriers had a significantly reduced risk of AD. In addition, the protective effect of rs2062323T on AD also applies to the European population. Cognitive decline was significantly slower in rs2062323T carriers in ADNI. Compared with the non‐carriers, the rs2062323T carriers showed significantly increased CSF sTREM2 levels.

Of note, the moderating effects of age on the association between rs2062323 and CSF sTREM2 were only detected in the Han Chinese population. The frequency of the rs2062323T varies between ethnic groups in the 1000 Genomes Project. The frequency is around 50% in the Asian populations, and about 30% in the US and European populations (http://feb2014.archive.ensembl.org/Homo_sapiens/Variation/Population?db=core;r=6:41239144‐41240144;v=rs2062323;vdb=variation;vf=1633078), and this difference may affect the association between rs2062323 and sTREM2. In addition, associations between rs2062323T and sTREM2 were significant in AD and non‐AD populations, suggesting that these associations may not be affected by diagnostic status.

The GTEx databases showed that rs2062323T was associated with high expression of the *TREM1*, *TREM2*, and *TREML1* genes in parietal brain regions. The TREM1, TREM2, and TREML1 belong to the TREM receptor family and can regulate inflammation by magnifying or inhibiting the toll‐like receptor‐induced signaling.^25^ Human TREM1 was an amplifier of acute inflammation and can link innate and adaptive immunity.^5^ TREM family genes were located at 6p21.1 in humans. Previous studies suggested that the 6p21.1 region is closely related to AD.^26^ Neuroinflammation is one of the critical pathogenic mechanisms of AD and is associated with selective neuronal vulnerability.^27^, ^28^ Microglia are immune cells residing in the central nervous system and play a critical regulatory role in maintaining brain homeostasis. Microglia (namely macrophages) are important mediators of neuroinflammation in AD.^29^ Previous studies have revealed associations between the TREM family genes (mainly *TREM1* and *TREM2*) and AD risk. Given the homology among the TREM family genes, the TREM region likely contains a number of distinct variations and genes that affect various aspects of AD risk.^9^ Previous research has demonstrated that the adaptor protein DAP12 facilitates the signaling of TREM1 and TREM2, which in turn activates myeloid cells.^5^

The TREM2 protein is present throughout the brain's white matter and is more abundant in the neocortex and hippocampus, but is absent from the cerebellum.^30^ Additionally, the TREM2 protein is primarily expressed in myeloid cells, including microglia, dendritic cells generated from monocytes, osteoblasts, and macrophages produced from bone marrow.^5^ Previous studies have shown that mutations and polymorphisms in the *TREM2* gene (rs75932628, rs143332484, rs142232675) are significantly associated with the risk of developing AD.^31^, ^32^ These AD‐associated risk polymorphisms have pathogenic consequences due to reduced TREM2 function.^30^ In AD patients, microglia processing of Aβ is an expansion and activation process, with activated microglia gathering near Aβ plaques and subsequently clearing Aβ. Thus, TREM2 deficiency can result in a significant decrease in the number of microglia surrounding the plaque.^33^ TREM2 does not bind to Aβ by itself. Notably, TREM2‐mediated microglia polarization, processing, and plaque encapsulation depend on the lipidation of Aβ. TREM2 can be activated by the lipid component of the lipoprotein complex. This specific mechanism gives TREM2 the ability to sense its surroundings and mediate the phagocytosis of dead neurons, myelin, and amyloid plaques.^33^ Decreased transcript levels of the *TREM2* gene may affect microglia activation and thus diminish the processing of dead neurons, myelin, and amyloid plaques, and maybe a potential pathway for TREM1 involvement in AD progression.

The sTREM2 is generated either by the proteolysis of TREM2 or by a selective shear of TREM2 lacking a transmembrane domain. The role and importance of sTREM2 are not yet fully understood. It has been shown that sTREM2 may be promoting microglial survival and stimulate the production of innate immune factors as well asTREM2 protein.^34^ Suárez‐Calvet et al. showed that CSF sTREM2 levels were significantly elevated in the early stages of AD, which may reflect a corresponding change in microglia activation status after neuronal degeneration.^22^ Heslegrave et al.’ study also revealed significantly elevated levels of CSF sTREM2 in AD, which may imply that CSF sTREM2 can be utilized to quantify the activation of glial cells.^35^ Notably, as AD progresses, the level of sTREM2 varies dynamically. CSF sTREM2 was correlated with markers of neuronal damage (CSF T‐tau and CSF p‐tau), suggesting that it could be used as a biomarker of neurodegeneration. In addition, sTREM2 was found to co‐localize with neurons and plaques in vivo, and sTREM2 may play a chemoattractant role in recruiting microglia to the vicinity of plaques.^36^, ^37^ The sTREM2 can also trigger microglia activation and induce an inflammatory response to protect microglia from apoptosis.^38^ In addition, sTREM2 has a protective effect against amyloid pathology and associated toxicity. The sTREM2 not only reduces a load of amyloid plaques to rescue functional defects in spatial memory, but also enhances microglia proliferation, migration, aggregation, and uptake and degradation of Aβ in the vicinity of amyloid plaques.^36^, ^38^ The *TREM1* gene intron variant rs2062323T is related to higher levels of sTREM2, suggesting that this variant may lessen the risk of AD through sTREM2‐mediated protection against amyloid pathology and associated toxicity.

Previous studies have shown that TREM2 is an important target for drug development. In 2019, Alector/abbvie claimed that they developed a monoclonal antibody (AL002) that specifically binds and activates TREM2 and thus improves AD pathology. In addition, a study from the University of Washington School of Medicine further validated the potential therapeutic effects of AL002c, a variant of AL002. Their results showed that long‐term administration of AL002c reduced filamentous plaques and neurodystrophy and alleviated microglia‐mediated inflammatory responses, which in turn improving symptoms.^39^ Given that rs2062323T is associated with high expression of *TREM2* and *sTREM2* genes, overexpression of rs2062323T by ligands or small molecules may be a potential route for drug discovery. In addition, the variant rs9357347C at 46 kb upstream of the TREM2 gene (between *TREM2* and *TREML2* genes) is associated with elevated expression levels of TREML1 and TREM2 and reduces the risk of developing AD.^40^ TREML1 plays a vital role in promoting vascular homeostasis and limiting inflammation.^41^, ^42^ The rs2062323T was also associated with higher levels of TREML1 and TREM2 expression, and this locus may have a protective effect on AD by enhancing vascular homeostasis and limiting neuroinflammation.

There are limitations to this study. First, because our study was an observational one, the causal relationship between rs2062323 and CSF sTREM2 needs to be confirmed further. Second, given that the level of sTREM2 varies dynamically, the protective effect of rs2062323T may only exist in a particular age group. Third, our AD diagnosis was made based on the NINCDS–ADRDA criteria, and the results may be more accurate if combined with biomarkers. Fourth, this study was conducted with modest sample sizes, and the generalizability of our conclusions might be restricted by the sources of studied populations. Further large‐scale community‐based longitudinal studies are warranted to validate these associations.

## 5 CONCLUSION

In conclusion, *TREM1* rs2062323 is an AD‐protective SNP that is linked to higher levels of CSF sTREM2. The rs2062323 may be involved in AD by regulating the expression of TREM1, TREML1, TREM2, and sTREM2. In future studies, using systems biology concepts to clarify the associations among TREM family members (TREM1, TREM2, and TREML1) in AD vulnerability may help to develop more effective therapeutic strategies.

## AUTHOR CONTRIBUTIONS

JTY and LT had full access to all of the data in the study and take responsibility for the integrity of the data and accuracy of the data analysis. Concept and design: JTY and LT. Acquisition, analysis, or interpretation of data: all authors. Drafting of the manuscript: ZTW, YF, SDC, YYH, and YHM. Critical revision of the manuscript for important intellectual content: All authors. Statistical analysis: ZTW and YF. Obtained funding: JTY and LT. Administrative, technical, or material support: YJW, JTY, and LT. All authors read and approved the final manuscript.

## FUNDING INFORMATION

This study was supported by grants from the National Natural Science Foundation of China (81771148, 82071201).

## CONFLICT OF INTEREST STATEMENT

The authors declare that they have no conflict of interest, financial, or otherwise.

## ETHICS APPROVAL

All data sources used in this study received approval from an ethics standards committee on human experimentation and the procedures used in this study adhere to the tenets of the Declaration of Helsinki. Written consent for genetic screening were obtained from all participants or their legal representatives. Their confidentiality was preserved according to the guidelines for studies of human subjects. About ADNI, data involved in the study came from the publicly open Alzheimer's disease neuroimaging initiative (ADNI) database.

## CONSENT TO PARTICIPATE

Informed written consent was obtained for all participants.

## Supporting information


Data S1.

## Data Availability

The data that support the findings of this study are available from the corresponding author upon reasonable request.
