# Supplementary material for: Association of rs2062323 in the TREM1 gene with Alzheimer's disease and cerebrospinal fluid‐soluble TREM2
Source: CNS Neurosci Ther. 2023 Feb 23;29(6):1657–66. doi: 10.1111/cns.14129 (PMC10173721; doi:10.1111/cns.14129)
Supplement: Supplementary file 1 — Data S1. [file CNS-29-1657-s001.docx]

**Additional Figure 1. Construction of linkage disequilibrium blocks and selection of tag-SNPs within the TREM1 genes (Han Chinese in Bejing)**

**
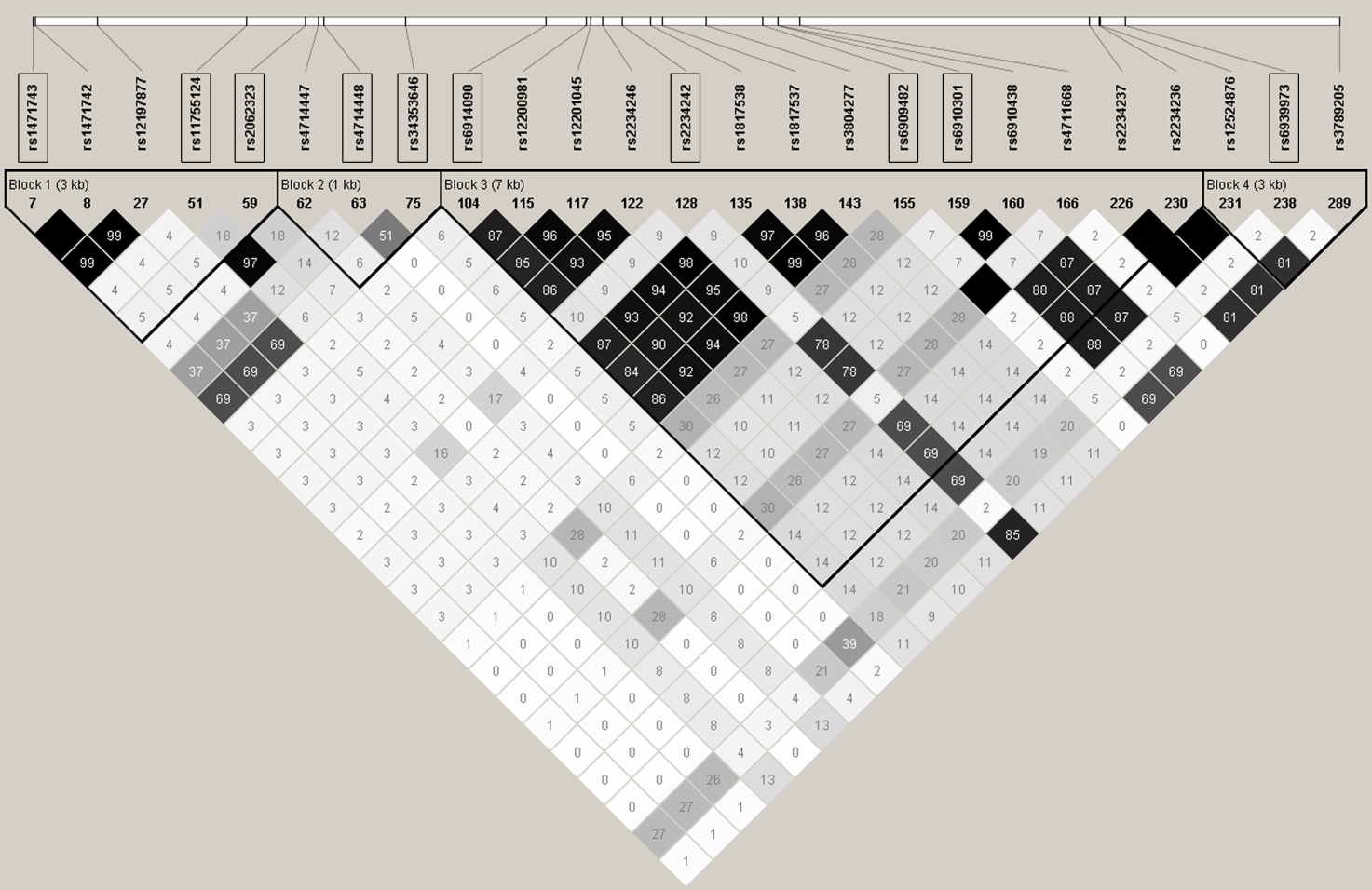
**

**Additional Figure 2. Construction of linkage disequilibrium blocks and selection of tag-SNPs within the TREM1 genes (Southern Han Chinese)**

**
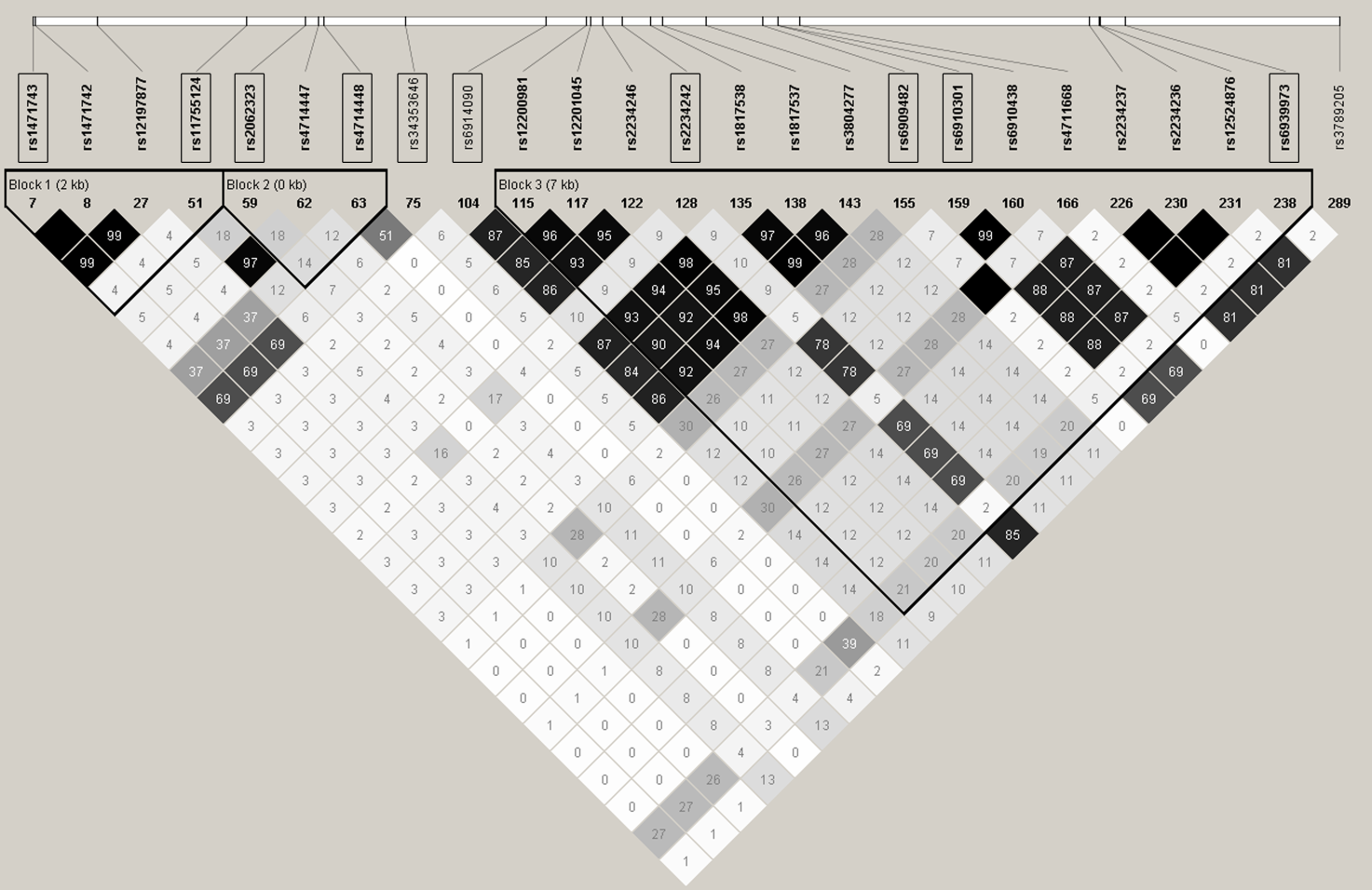
**

**Additional Figure 3. Construction of linkage disequilibrium blocks and selection of tag-SNPs within the TREM1 genes (European)**

**
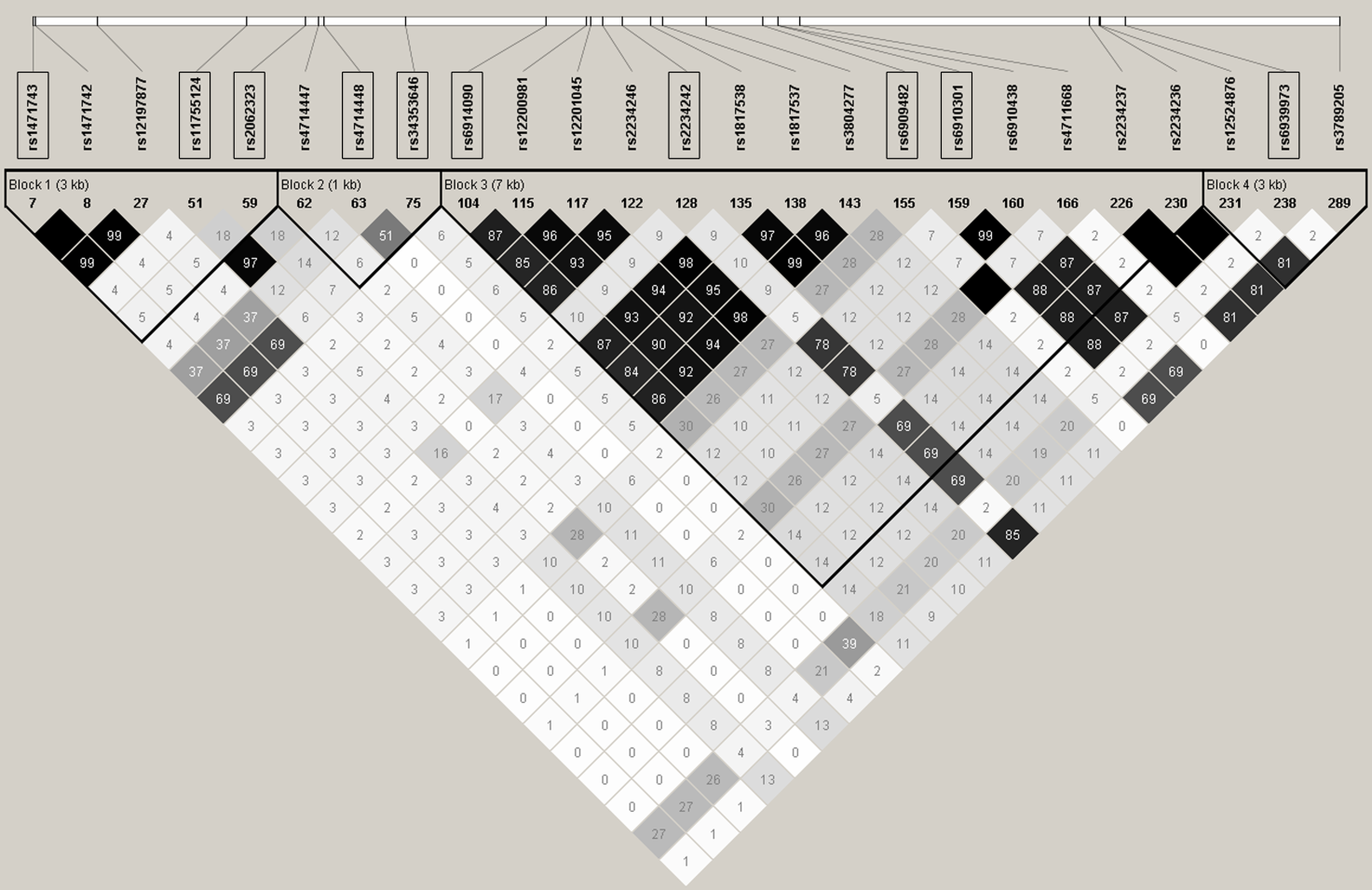
**

**Additional Figure 4. Multi-tissue eQTL of *TREM1* gene expression**


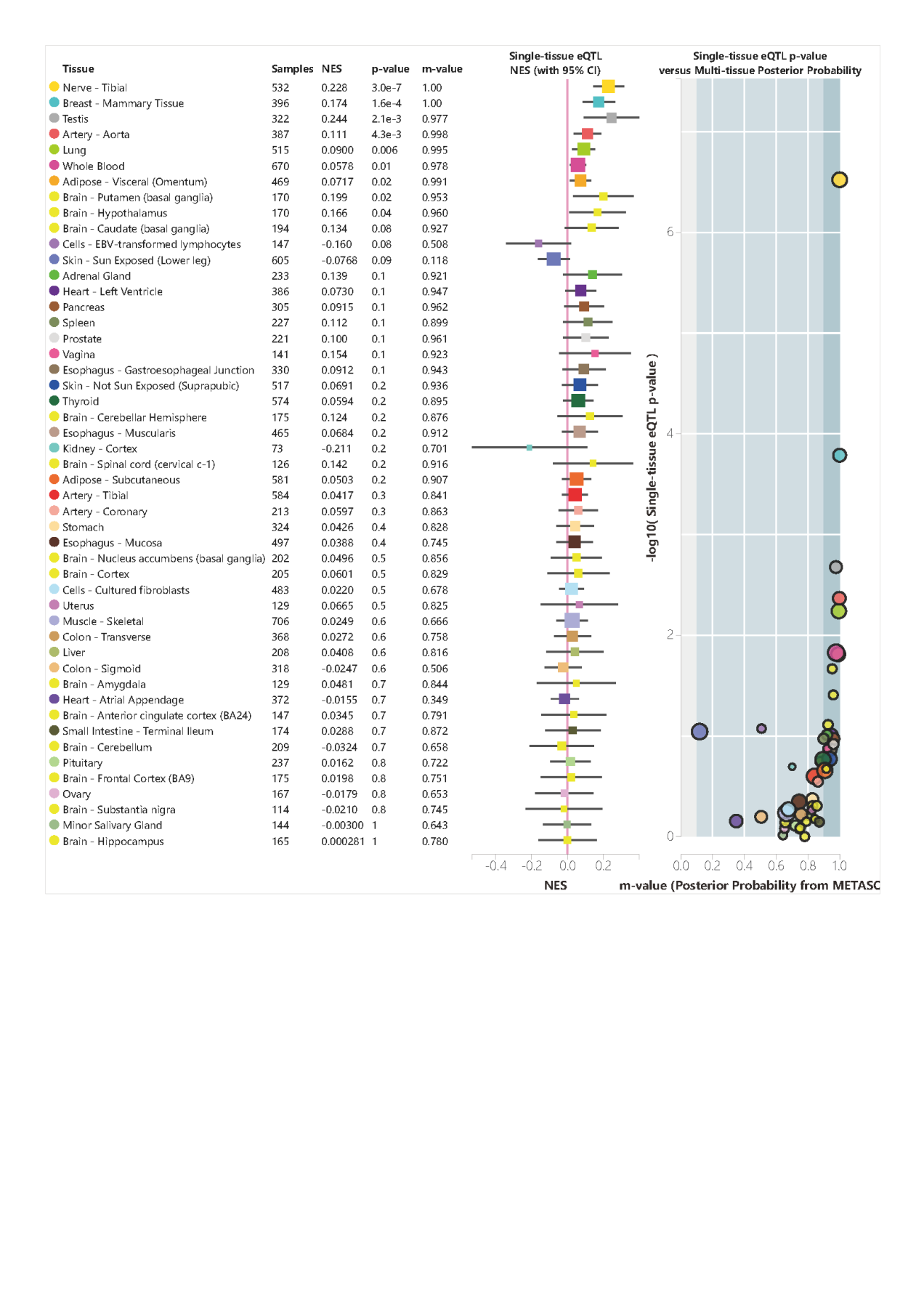


**Additional Figure 5. Multi-tissue eQTL of *TREM2* gene expression**

**
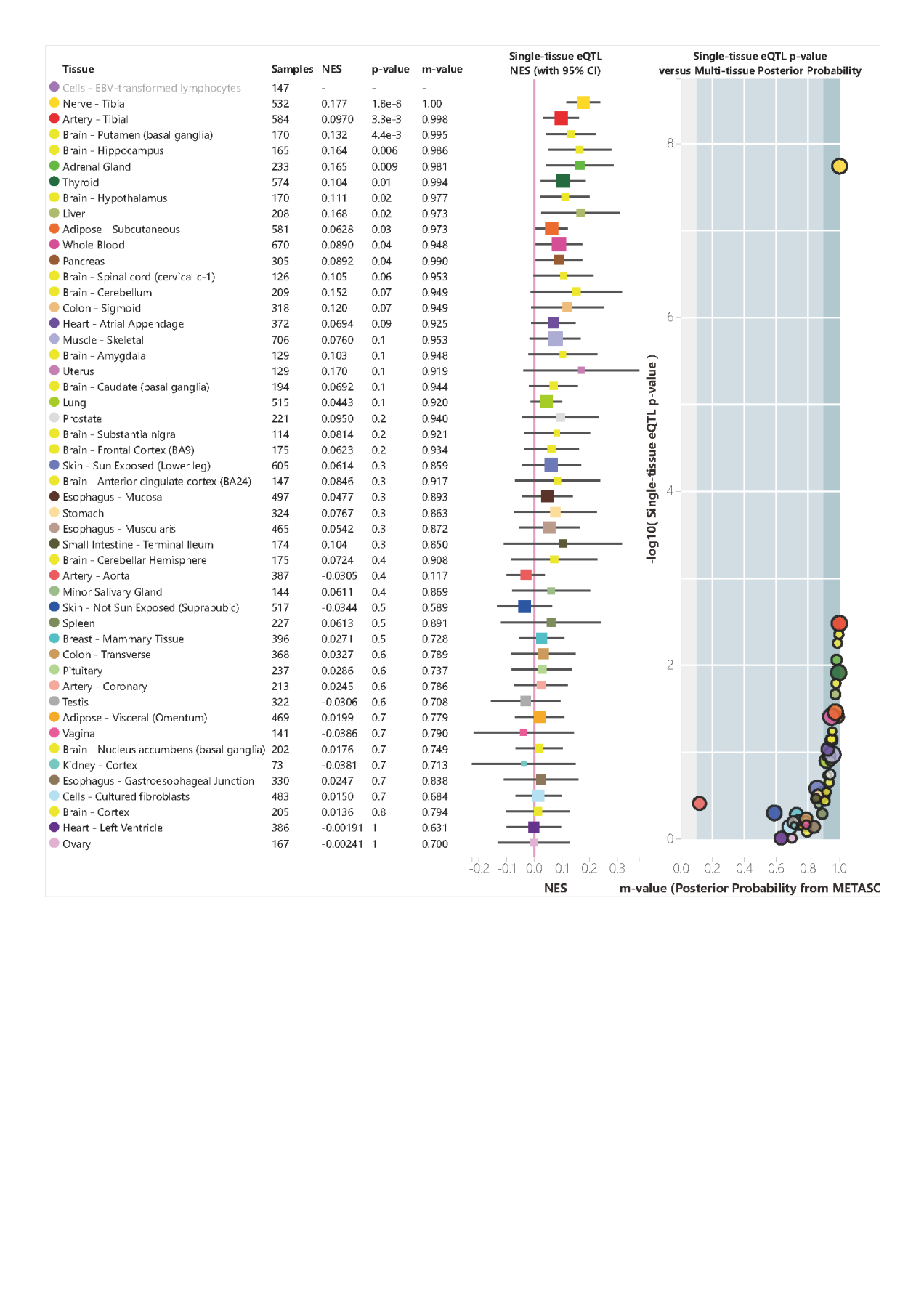
**

**Additional Figure 6. Multi-tissue eQTL of *TREML1* gene expression**

**
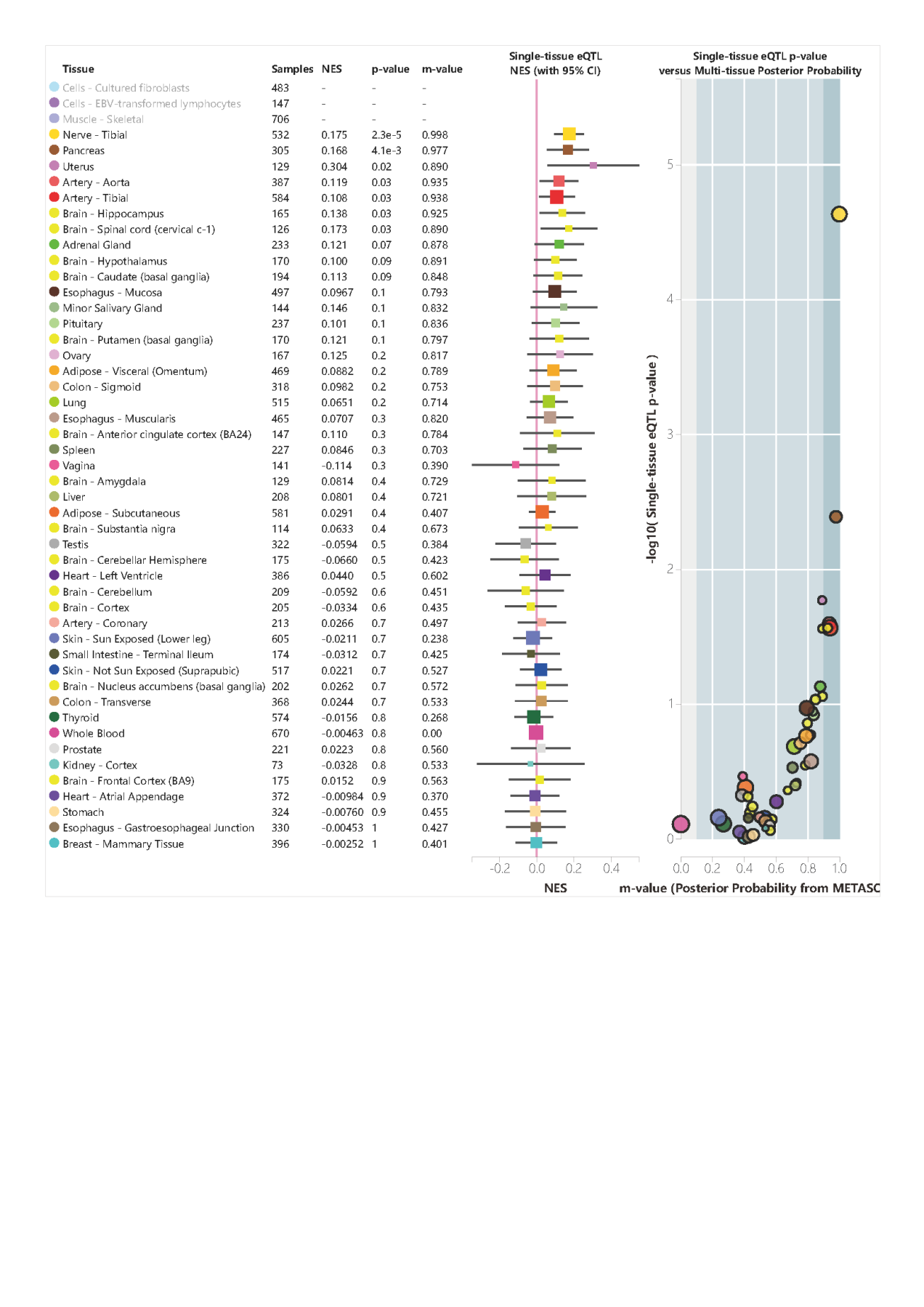
**

**Additional table 1. Genotype distributions of the tag-SNPs and their associations with the risk of sporadic Alzheimer’s disease**

| Gene | **Tag-SNPs** | **Location^a^** | **Allele (major/minor)** | **MAF** | **MAF in CHB^b^** | **P value of HWE** |
| --- | --- | --- | --- | --- | --- | --- |
| *TREM1* | rs1471743 | Chr 6:41235879 intron | A/G | 0.118 | 0.123 | 1.00 |
| *TREM1* | rs11755124 | Chr 6:41238833 intron | C/A | 0.353 | 0.293 | 0.140 |
| *TREM1* | rs2062323 | Chr 6:41239644 intron | C/T | 0.476 | 0.313 | 0.019 |
| *TREM1* | rs4714448 | Chr 6:41239906 intron | A/G | 0.120 | 0.235 | 0.918 |
| *TREM1* | rs34353646 | Chr 6:41241027 intron | A/G | 0.118 | 0.143 | 0.508 |
| *TREM1* | rs6914090 | Chr 6:41242972 exon | T/C | 0.273 | 0.426 | 0.119 |
| *TREM1* | rs2234242 | Chr 6:41244020 intron | G/A | 0.267 | 0.126 | 0.101 |
| *TREM1* | rs6909482 | Chr 6:41245953 intron | C/A | 0.460 | 0.290 | 0.082 |
| *TREM1* | rs6910301 | Chr 6:41246166 intron | G/A | 0.419 | 0.155 | 0.037 |
| *TREM1* | rs6939973 | Chr 6:41250961 intron | A/G | 0.116 | 0.122 | 0.222 |
| ^a^ The genomic coordinates were shown based on the human assembly GRCh37, and relative locations were referred to the NGFR gene. ^b^ Data were obtained from HapMap database for Chinese Han in Beijing (CHB). Abbreviations: MAF, minor allele frequency; HWE, Hardy-Weinberg equilibrium. | | | | | | |

**Additional table 2. Genotype distributions of the tag-SNPs**

| **Tag-SNPs** | **Genotypes** | **Control group** | **AD group** |
| --- | --- | --- | --- |
| rs1471743 | A/A | 676 (0.777) | 848 (0.784) |
|  | A/G | 183 (0.21) | 212 (0.196) |
|  | G/G | 11 (0.013) | 21 (0.019) |
| rs11755124 | C/C | 385 (0.443) | 423 (0.391) |
|  | C/A | 391 (0.449) | 517 (0.478) |
|  | A/A | 94 (0.108) | 141 (0.13) |
| rs2062323 | C/C | 207 (0.238) | 316 (0.292) |
|  | C/T | 450 (0.517) | 547 (0.506) |
|  | T/T | 213 (0.245) | 218 (0.202) |
| rs4714448 | A/A | 678 (0.779) | 840 (0.777) |
|  | A/G | 182 (0.209) | 215 (0.199) |
|  | G/G | 10 (0.011) | 26 (0.024) |
| rs34353646 | A/A | 684 (0.786) | 844 (0.781) |
|  | A/G | 176 (0.202) | 211 (0.195) |
|  | G/G | 10 (0.011) | 26 (0.024) |
| rs6914090 | T/T | 471 (0.541) | 557 (0.515) |
|  | T/C | 344 (0.395) | 436 (0.403) |
|  | C/C | 55 (0.063) | 88 (0.081) |
| rs2234242 | G/G | 482 (0.554) | 567 (0.525) |
|  | G/A | 316 (0.363) | 448 (0.414) |
|  | A/A | 72 (0.083) | 66 (0.061) |
| rs6909482 | C/C | 245 (0.282) | 318 (0.294) |
|  | C/A | 426 (0.49) | 556 (0.514) |
|  | A/A | 199 (0.229) | 207 (0.191) |
| rs6910301 | G/G | 469 (0.539) | 548 (0.353) |
|  | G/A | 324 (0.372) | 457 (0.294) |
|  | A/A | 77 (0.089) | 548 (0.353) |
| rs6939973 | A/A | 682 (0.784) | 851 (0.787) |
|  | A/G | 179 (0.206) | 206 (0.191) |
|  | G/G | 9 (0.01) | 24 (0.022) |

**Additional table 3. Multiple regressions results for associations of CSF AD biomarkers with rs2062323**

| **Genetic modelsa^α^** | **CSF sTREM2** | | |  | **CSF Aβ42** | | |  | **CSF P-tau** | | |  | **CSF T-tau** | | |
| --- | --- | --- | --- | --- | --- | --- | --- | --- | --- | --- | --- | --- | --- | --- | --- |
|  | **n** | **Mean ± SD** | **p-value** |  | **n** | **Mean ± SD** | **p-value** |  | **n** | **Mean ± SD** | **p-value** |  | **n** | **Mean ± SD** | **p-value** |
| **Codominant** |  |  |  |  |  |  |  |  |  |  |  |  |  |  |  |
| rs2062323^CC^ | 96 | 18085.69 ± 6717.40 |  |  | 85 | 287.54 ± 157.51 |  |  | 104 | 43.46 ± 13.61 |  |  | 102 | 197.35 ± 77.80 |  |
| rs2062323^CT^ | 219 | 19021.05 ± 6953.55 | 0.1914 |  | 204 | 270.13 ± 141.87 | 0.4161 |  | 244 | 43.63 ± 13.38 | 0.7649 |  | 221 | 202.28 ± 89.31 | 0.6235 |
| rs2062323^TT^ | 106 | 18549.34 ± 7069.10 | 0.6772 |  | 95 | 242.08 ± 126.19 | 0.0624 |  | 116 | 41.94 ± 14.19 | 0.3395 |  | 99 | 188.94 ± 118.81 | 0.0699 |
| **Dominant** |  |  |  |  |  |  |  |  |  |  |  |  |  |  |  |
| rs2062323^CC^ | 96 | 18085.69 ± 6717.395 | 0.2760 |  | 85 | 287.54 ± 157.51 | 0.1900 |  | 104 | 43.46 ± 13.61 | 0.8664 |  | 102 | 197.35 ± 77.80 | 0.7170 |
| rs2062323^CT/TT^ | 325 | 18867.20 ± 6984.039 |  |  | 299 | 261.22 ± 137.49 |  |  | 360 | 43.08 ± 13.65 |  |  | 320 | 198.15 ± 99.37 |  |
| **Additive** |  |  |  |  |  |  |  |  |  |  |  |  |  |  |  |
| rs2062323^CC^ (0) | 96 | 18085.69 ± 6717.40 | 0.7055 |  | 85 | 287.54 ± 157.51 | 0.0604 |  | 104 | 43.46 ± 13.61 | 0.3207 |  | 102 | 197.35 ± 77.80 | 0.0734 |
| rs2062323^CT^ (1) | 219 | 19021.05 ± 6953.55 |  |  | 204 | 270.13 ± 141.87 |  |  | 244 | 43.63 ± 13.38 |  |  | 221 | 202.28 ± 89.31 |  |
| rs2062323^TT^ (2) | 106 | 18549.34 ± 7069.10 |  |  | 95 | 242.08 ± 126.19 |  |  | 116 | 41.94 ± 14.19 |  |  | 99 | 188.94 ± 118.81 |  |

^α^Assuming M represents major allele and m represents minor allele, each genetic model can be described as follows: codominant: M/m vs M/M and m/m vs M/M, two OR values were listed from top to bottom in corresponding columns; dominant: (m/m + M/m) vs M/M; additive: m/m and M/m were weighed 2 and 1 respectively to M/M.

All models were adjusted by age, sex, APOE ε4, education (years) and MMSE score.

Abbreviations: SNP, Single nucleotide polymorphism; SD, standard deviation; CSF, cerebrospinal fluid; AD, Alzheimer’s disease; AV45, 18F-AV45 amyloid-PET; sTREM2, soluble TREM2; Aβ, amyloid-beta.

**Additional table 4. Moderating effects of age, sex, and APOE4 on the association between rs2062323 and CSF biomarkers.**

| **Variable or interaction** | **CSF sTREM2** | |  | **CSF Aβ42** | |  | **CSF P-tau** | |  | **CSF T-tau** | |
| --- | --- | --- | --- | --- | --- | --- | --- | --- | --- | --- | --- |
|  | **B** | **p-Value** |  | **B** | **p-Value** |  | **B** | **p-Value** |  | **B** | **p-Value** |
| **Model for age effect** |  |  |  |  |  |  |  |  |  |  |  |
| rs2062323^CT^ × age | -0.0234 | 0.0889 |  | 0.0030 | 0.8290 |  | -0.0119 | 0.3452 |  | -0.0029 | 0.8198 |
| rs2062323^TT^ × age | -0.0308 | **0.0436** |  | 0.0010 | 0.9480 |  | -0.0094 | 0.5135 |  | 0.0076 | 0.6016 |
| **Model for sex effect** |  |  |  |  |  |  |  |  |  |  |  |
| rs2062323^CT^ × sex | -0.1992 | 0.4085 |  | 0.1553 | 0.5500 |  | -0.3729 | 0.0954 |  | -0.3891 | 0.0841 |
| rs2062323^TT^ × sex | -0.4225 | 0.1312 |  | 0.3282 | 0.2794 |  | -0.3962 | 0.1259 |  | -0.2403 | 0.3691 |
| **Model for *APOE4* effect** |  |  |  |  |  |  |  |  |  |  |  |
| rs2062323^CT^ × *APOE4* | 0.2173 | 0.5185 |  | 0.1671 | 0.6430 |  | 0.3787 | 0.2408 |  | 0.0202 | 0.9494 |
| rs2062323^TT^ × *APOE4* | 0.6769 | 0.0546 |  | -0.1587 | 0.6790 |  | 0.3683 | 0.2713 |  | 0.2229 | 0.5170 |

Adjusted for age, gender, education, APOE ε4 status.

Abbreviations: Aβ, amyloid-beta; B, unstandardized regression coefficient; OR, odds ratio.

**Additional table 5. Baseline demographic characteristics of participants included.**

| **Characteristic** | **Non-demented (n=676)** | **AD (n=166)** | **P** |
| --- | --- | --- | --- |
| Age (years, mean ± SD) | 72.814 ± 6.951 | 74.838 ± 8.243 | 0.001 |
| Sex (female, %) | 282 (41.72) | 67 (40.36) | 0.751 |
| Education (years, mean ± SD) | 16.27 ± 2.796 | 15.48 ± 2.845 | 0.001 |
| *APOE ε4* (yes, %) | 289 (42.75) | 115 (69.28) | < 0.001 |
| MMSE score (mean ± SD) | 28.23 ± 1.71 | 23.21 ± 2.012 | < 0.001 |
| ADASQ4 score (mean ± SD) | 4.56 ± 2.628 | 8.69 ± 1.451 | < 0.001 |
| RAVLT_learning score (mean ± SD) | 4.85 ± 2.599 | 1.68 ± 1.69 | < 0.001 |
| CSF sTREM2 (pg/ml) | 4026.666 ± 1889.233 | 4286.119 ± 2064.263 | 0.120 |

ANOVA, non-parametric Kruskal-Wallis H test, and Chi-squared test were used to compare the baseline demographic and clinical

characteristics. Abbreviations: SD, standard deviation; MMSE, Mini-Mental State Exam; ADAS, Alzheimer's Disease Assessment

Scale; RAVLT, Rey Auditory Verbal Learning Test ; CSF, cerebrospinal fluid; AD, Alzheimer’s disease.

**Additional table 6. Moderating effects of age, sex, and APOE4 on the association between rs2062323 and CSF sTREM2.**

| **Variable or interaction** | **CSF sTREM2 (non-demented)** | | |  |  | **CSF sTREM2 (AD)** | |
| --- | --- | --- | --- | --- | --- | --- | --- |
|  | **n** | **B** | **p-Value** |  | n | **B** | **p-Value** |
| **Model for age effect** |  |  |  |  |  |  |  |
| rs2062323^CT/TT^ | 676 | 1.009 | 0.204 |  | 166 | -0.638 | 0.641 |
| rs2062323^CT/TT^ × age |  | -0.011 | 0.299 |  |  | 0.013 | 0.490 |
| **Model for sex effect** |  |  |  |  |  |  |  |
| rs2062323^CT/TT^ | 676 | 0.121 | 0.301 |  | 166 | 0.629 | 0.007 |
| rs2062323^CT/TT^ × sex |  | 0.113 | 0.457 |  |  | -0.553 | 0.068 |
| **Model for *APOE4* effect** |  |  |  |  |  |  |  |
| rs2062323^CT/TT^ | 676 | 0.070 | 0.481 |  | 166 | 0.407 | 0.132 |
| rs2062323^CT/TT^ × *APOE4* |  | 0.276 | 0.070 |  |  | -0.152 | 0.638 |

Adjusted for age, sex, APOE ε4 status, and clinical diagnosis.

Abbreviations: Aβ, amyloid-beta; B, unstandardized regression coefficient; OR, odds ratio.

**Additional table 7. Baseline demographic characteristics of participants included.**

| **Characteristic** | **Non-demented (n=518)** | **AD (n=135)** | **P** |
| --- | --- | --- | --- |
| Age (years, mean ± SD) | 72.621 ± 6.8141 | 75.164 ± 7.9909 | < 0.001 |
| Sex (female, %) | 212 (33.97) | 53 (32.32) | 0.725 |
| Education (years, mean ± SD) | 16.19 ± 2.769 | 15.48 ± 2.807 | 0.008 |
| *APOE ε4* (yes, %) | 228 (36.54) | 91 (55.49) | < 0.001 |
| MMSE score (mean ± SD) | 28.21 ± 1.715 | 23.13 ± 2.018 | < 0.001 |
| ADASQ4 score (mean ± SD) | 4.69 ± 2.65 | 8.61 ± 1.486 | < 0.001 |
| RAVLT_learning score (mean ± SD) | 4.76 ± 2.661 | 1.63 ± 1.644 | < 0.001 |
| CSF sTREM2 (WU) (pg/ml) | 2455.619 ± 701.324 | 2502.494 ± 708.336 | 0.490 |

ANOVA, non-parametric Kruskal-Wallis H test, and Chi-squared test were used to compare the baseline demographic and clinical

characteristics. Abbreviations: SD, standard deviation; MMSE, Mini-Mental State Exam; ADAS, Alzheimer's Disease Assessment

Scale; RAVLT, Rey Auditory Verbal Learning Test ; CSF, cerebrospinal fluid; AD, Alzheimer’s disease.

| **Additional table 8. Multiple regressions results for associations of CSF sTREM2 (WU) with rs2062323 in ADNI** | | | | | | | |
| --- | --- | --- | --- | --- | --- | --- | --- |
| **Genetic modelsa^α^** | **CSF sTREM2 (non-demented)** | | |  | **CSF sTREM2 (AD)** | | |
|  | **n** | **Mean ± SD** | **β, p-value** |  | **n** | **Mean ± SD** | **β, p-value** |
| **Codominant** |  |  |  |  |  |  |  |
| rs2062323^CC^ | 253 | 2368.54 ± 664.875 | reference |  | 70 | 2400.13 ± 646.635 | reference |
| rs2062323^CT^ | 218 | 2537.84 ± 703.664 | **0.27, 0.0034** |  | 53 | 2561.25 ± 695.263 | 0.22, 0.1943 |
| rs2062323^TT^ | 47 | 2543 ± 830.049 | 0.25, 0.1134 |  | 12 | 2840.09 ± 998.242 | 0.54, 0.0784 |
| **Dominant** |  |  |  |  |  |  |  |
| rs2062323^CC^ | 253 | 2368.54 ± 664.875 | reference |  | 70 | 2400.13 ± 646.635 | reference |
| rs2062323^CT/TT^ | 265 | 2538.76 ± 725.979 | **0.26, 0.0024** |  | 65 | 2612.73 ± 758.889 | 0.28, 0.0886 |
| **Additive** |  |  |  |  |  |  |  |
| rs2062323^CC^ (0) | 253 | 2368.54 ± 664.875 | **0.18, 0.0070** |  | 70 | 2400.13 ± 646.635 | **0.25, 0.0490** |
| rs2062323^CT^ (1) | 218 | 2537.84 ± 703.664 |  |  | 53 | 2561.25 ± 695.263 |  |
| rs2062323^TT^ (2) | 47 | 2543 ± 830.049 |  |  | 12 | 2840.09 ± 998.242 |  |

^α^Assuming M represents major allele and m represents minor allele, each genetic model can be described as follows:

codominant: M/m vs M/M and m/m vs M/M, two OR values were listed from top to bottom in corresponding columns; dominant: (m/m + M/m) vs M/M;

additive: m/m and M/m were weighed 2 and 1 respectively to M/M.

All models were adjusted by age, sex, APOE ε4, education (years) and MMSE score. Later-life (> 65 y) versus Mid-life (≤65 y).

^β^Abbreviations: SNP, Single nucleotide polymorphism; SD, standard deviation; CSF, cerebrospinal fluid; AD, Alzheimer’s disease; AV45, 18F-AV45 amyloid-PET;

sTREM2, soluble TREM2; Aβ, amyloid-beta.

**Additional table 9. Moderating effects of age, sex, and APOE4 on the association between rs2062323 and CSF sTREM2 (WU).**

| **Variable or interaction** | **CSF sTREM2 (non-demented)** | | |  |  | **CSF sTREM2 (AD)** | |
| --- | --- | --- | --- | --- | --- | --- | --- |
|  | **n** | **B** | **p-Value** |  | n | **B** | **p-Value** |
| **Model for age effect** |  |  |  |  |  |  |  |
| rs2062323^CT/TT^ | 518 | 0.838 | 0.367 |  | 135 | -0.252 | 0.872 |
| rs2062323^CT/TT^ × age |  | -0.008 | 0.533 |  |  | 0.007 | 0.731 |
| **Model for sex effect** |  |  |  |  |  |  |  |
| rs2062323^CT/TT^ | 518 | 0.072 | 0.592 |  | 135 | 0.447 | 0.091 |
| rs2062323^CT/TT^ × sex |  | 0.322 | 0.064 |  |  | -0.273 | 0.419 |
| **Model for *APOE4* effect** |  |  |  |  |  |  |  |
| rs2062323^CT/TT^ | 518 | 0.265 | 0.021 |  | 135 | 0.286 | 0.323 |
| rs2062323^CT/TT^ × *APOE4* |  | -0.007 | 0.969 |  |  | -0.007 | 0.984 |

Adjusted for age, sex, APOE ε4 status, and clinical diagnosis.

Abbreviations: Aβ, amyloid-beta; B, unstandardized regression coefficient; OR, odds ratio.
